# Antiviral Activity of Pyrazolopyrimidine and Triazolopyrimidine Derivatives Against SARS-CoV-2 In Vitro: Identifying PZP25 as a Promising Scaffold

**DOI:** 10.3390/pathogens15030324

**Published:** 2026-03-18

**Authors:** Saiqa Sardar, Jessica S. C. C. Martins, Thiago C. Sousa, Andreon S. M. Silva, Marcelo A. Pinto, Flávia F. Silveira, Thais B. Silva, Rodolfo R. F. França, Luiz C. S. Pinheiro, Nubia Boechat, Marilda M. Siqueira, Aline R. Matos, Leonardo J. M. Carvalho

**Affiliations:** 1 Laboratório de Vírus Respiratórios, Exantemáticos, Enterovírus e Emergências Virais, Instituto Oswaldo Cruz (IOC-Fiocruz), Rio de Janeiro 21040-360, RJ, Brazil; saiqasardar@aluno.fiocruz.br (S.S.); jessica.martins@ioc.fiocruz.br (J.S.C.C.M.); thiagosousarj@gmail.com (T.C.S.); mmsiq@ioc.fiocruz.br (M.M.S.); aline.matos@ioc.fiocruz.br (A.R.M.); 2 Laboratório de Pesquisa em Malária, Instituto Oswaldo Cruz (IOC-Fiocruz), Rio de Janeiro 21045-900, RJ, Brazil; 3 Laboratório de Desenvolvimento Tecnológico em Virologia, Instituto Oswaldo Cruz (IOC-Fiocruz), Rio de Janeiro 21040-360, RJ, Brazil; andreon_sms@hotmail.com (A.S.M.S.); marcelop@ioc.fiocruz.br (M.A.P.); 4 Laboratório de Síntese de Fármacos, Instituto de Tecnologia em Fármacos (Farmanguinhos-Fiocruz), Rio de Janeiro 21041-250, RJ, Brazil; flaviafernandes_jo23@hotmail.com (F.F.S.); britosilvathais18@gmail.com (T.B.S.); rodolfo.franca@fiocruz.br (R.R.F.F.); luiz.pinheiro@uerj.br (L.C.S.P.); nubia.boechat@fiocruz.br (N.B.); 5 Departamento de Ciências, Universidade do Estado do Rio de Janeiro (UERJ), Sao Goncalo 24435-005, RJ, Brazil

**Keywords:** SARS-CoV-2, COVID-19, antiviral, pyrazolopyrimidines, triazolopyrimidines, drug discovery, chemical scaffold

## Abstract

Prior molecular docking and dynamics studies indicated a pyrazolopyridine–sulfonamide derivative (L87/PPS2, or simply PPS2) as a potential interactant with SARS-CoV-2 protein targets. The in vitro anti-SARS-CoV-2 activity and cytotoxicity profile of PPS2 were screened alongside a series of pyrazolopyrimidine (PZP) and triazolopyrimidine (TZP) derivatives. PPS2 demonstrated only partial inhibition of SARS-CoV-2 growth in Vero E6 cells at 100 µM. Crucially, however, four out of five PZPs and eight out of fourteen TZPs exhibited potent in vitro inhibitory activity against SARS-CoV-2 at 100 µM, with none of the tested compounds displaying cytotoxicity against Vero E6 cells at this concentration. Further characterization of one compound, PZP25, revealed an inhibitory concentration (IC50) of 8.2 µM, combined with low cytotoxicity (CC50 > 800 µM), yielding a selectivity index greater than 100. Time of addition assays indicated that PZP25’s antiviral effects were most pronounced when administered post-infection. While cellular pre-treatment provided a partial reduction in virus growth, modest virucidal activity was also observed at warmer temperatures (20 °C and 37 °C). Collectively, our findings demonstrate that PZP and TZP derivatives possess potent inhibitory activity of SARS-CoV-2 replication in vitro and highlight such compounds as promising chemical scaffolds for the development of novel antiviral agents targeting coronaviruses.

## 1. Introduction

Despite significant global efforts in vaccine development and therapeutic advancements, coronavirus disease 2019 (COVID-19), caused by the severe acute respiratory syndrome coronavirus 2 (SARS-CoV-2), continues to pose a major public health challenge, resulting in millions of deaths worldwide [1] and highlighting the persistent need for novel antiviral strategies. SARS-CoV-2 infects host cells primarily through its spike protein binding to ACE2 receptors, with host proteases like TMPRSS2 facilitating viral entry. This process initiates a complex pathological cascade characterized by viral replication, immune evasion, cytokine storm, alveolar damage, microvascular thrombosis, and endothelial injury, collectively leading to multi-organ damage and severe clinical complications [2].

The COVID-19 public health crisis triggered the world into rapid advances in the development of therapeutic, diagnostic, and digital health technologies. Initially, COVID-19 treatment focused on supportive care for patients with severe respiratory distress, including oxygen supplementation and mechanical ventilation for more severe cases [3]. Since then, there has been a substantial evolution in the development of various preventive and therapeutic options, including vaccines, antiviral agents, monoclonal antibodies, and immunomodulators, aimed at combating the disease and the crisis it generated [4,5]. While numerous COVID-19 vaccines have significantly reduced severe disease and mortality across various platforms (mRNA, viral vectors, inactivated virus, and protein subunits), the current landscape still lacks highly effective therapeutic interventions [6].

Several viral components represent potential targets for therapeutic intervention, including both structural and nonstructural proteins, such as the S protein, the 3C-like cysteine protease [3CLpro], papain-like cysteine protease, RNA-dependent RNA polymerase (RdRp), and helicase/NTPase [7]. Remdesivir, the first FDA-approved antiviral for COVID-19, inhibits viral replication by targeting the RNA-dependent RNA polymerase but provides only modest clinical benefits [8]. Oral antivirals like molnupiravir and Paxlovid have expanded outpatient treatment options and reduced disease progression, yet no drug has fully resolved severe outcomes [9,10]. Immunomodulatory therapies such as cytokine inhibitors, corticosteroids, and even angiotensin receptor blockers and angiotensin-converting enzyme inhibitors have been used to manage hyperinflammation [11,12,13]. However, the availability of new effective drugs to treat COVID-19 remains a necessity in view of the continued transmission of SARS-CoV-2, including the ongoing emergence of viral variants of concern, which may be more prone to cause severe disease. Recently, an in silico study reported that a compound named L87 showed great potential to be used against SARS-CoV-2 [14]. The SARS-CoV-2 main protease 3CLpro was shown to interact well with the binding site of L87, with a high binding free energy score of about −7.607 kcal/mol, suggesting an interaction more stable than other molecules, such as the clinical medications remdesivir and arbidol. The L87 molecule creates three major interactions with the protease’s active site residues (THR26, GLU166, and GLY143), with bonding distances confirming the strength of these interactions. Molecular dynamics simulations indicated that the L87–protease complex is stable throughout time. The compound L87 evaluated by Daoud et al. [14] was previously described among a series of pyrazolopyridine–sulfonamide derivatives, which were designed, synthesized, and tested for antimalarial activity by our group and showed inhibitory activity against malaria parasites [15]. This molecule (originally named pyrazolopyridine–sulfonamide compound 2, or PPS2) was, therefore, selected for assessment of its in vitro activity against SARS-CoV-2 in the present study. Beyond L87/PPS2, the broader class of pyrimidine ring system derivatives, specifically triazolopyrimidines (TZPs) and pyrazolopyrimidines (PZPs), has garnered significant attention due to their diverse biological activities, including antiviral, anti-inflammatory, antimicrobial, antidiabetic, anti-Alzheimer’s, and antitumoral properties [16,17,18,19]. Given their structural relationship to L87/PPS2 and a proven track record of strong in vitro inhibitory activity against Plasmodium falciparum [20], a comprehensive series of TZP and PZP derivatives was also strategically selected for in vitro screening against SARS-CoV-2 in the present study. These compounds collectively represent promising chemical scaffolds and lead candidates for the development of novel COVID-19 therapeutics. Considering the ongoing emergence of viral variants, the findings of this study may offer critical insights for designing effective therapeutic strategies to combat COVID-19.

## 2. Materials and Methods

### 2.1. Study Compounds

Compound PPS2 and a series of 14 TZP and 5 PZP derivatives were used in this study for their antiviral activity. These compounds were synthesized and supplied by Farmanguinhos, Fiocruz, Brazil. Some of these derivatives are in their hydrochloride form. The chemical structures are described in Table 1 and Table 2 and have been previously published [15,20]. The compounds were purified by chromatography on TLC glass plates (Merck Silica gel 60 RP-18 F254s) in chloroform/methanol (9.5:0.5) or by recrystallization from methanol/water (2:1). The structural elucidation of the compounds was carried out by nuclear magnetic resonance spectroscopy (Bruker Avance, Bruker Co., Ltd., Billerica, MA, USA) and high-resolution mass spectrometry (Micromass/Waters ZQ-4000, Waters Co., Ltd., Milford, MA, USA). Their purities were determined by HPLC (Bruker Daltonics MicroTOF, Bruker Co., Ltd., Billerica, MA, USA), in which all compounds showed area values greater than 95%. The data had been previously published [15,20]. The molecules were provided in lyophilized form, and stock solutions were prepared by dissolving them in anhydrous dimethyl sulfoxide (DMSO) (Merck KGaA, Darmstadt, Germany). For comparative evaluation, the antiviral agents remdesivir (RDV) and nirmatrelvir (NMV) (MedchemExpress, Monmouth Junction, NJ, USA) were used. Due to their photosensitive nature, all the tested compounds were protected from light throughout handling to preserve their biochemical integrity prior to antiviral testing against SARS-CoV-2.

### 2.2. Cell Culture

For the in vitro model for antiviral evaluation, Vero E6 and Vero CCL-81 cell lines were used, established from kidney epithelial cells of the African green monkey (genus *Chlorocebus*, formerly *Cercopithecus aethiops*), which are permissive for SARS-CoV-2 infection and replication [21]. Dulbecco’s Modified Eagle Medium (DMEM), containing L-Glutamine (3.9 mM) and D-glucose (4.5 g/L), supplemented with 10% Fetal Bovine Serum (FBS), Streptomycin 100× Penicillin solution (with final concentrations of 100 μg/mL and 100 U/mL, respectively) was used as cell culture medium to support optimal growth and viability. Both cell lines were incubated at 37 °C and 5% CO_2_ with 95% relative humidity. In addition, routine screening of cells was performed for mycoplasma contamination through polymerase chain reaction (PCR) to ensure the reliability and integrity of the cell culture system.

### 2.3. Viral Isolate

The SARS-CoV-2 strain used in this study was isolated in 2020 from a clinical specimen (combined nasopharyngeal and oropharyngeal swab) obtained from a confirmed COVID-19 case in Rio de Janeiro, Brazil. The virus was propagated on Vero E6 cells, and the resulting viral seeds were propagated through two successive passages to produce a working stock in Vero E6 cell cultures. The virus identity was confirmed (isolate Brazil/RJ-314/2020, deposited on the GISAID platform under accession number EPI_ISL ID 427294 (https://www.epicov.org/)) as the original Wuhan lineage (Pango B.1.1.33 lineage). Viral titers were determined as plaque-forming units per milliliter of viral culture (PFU/mL). All procedures related to viral isolation (as well as all subsequent experimentation with the isolate) were conducted in a Biosafety Level 3 (BSL-3) facility of the institutional multi-user platform at Fiocruz. The experimental procedure was carried out following institutional biosafety protocols and in compliance with national and international (WHO) guidelines for work with SARS-CoV-2.

### 2.4. Viral Inhibition Assay

All incubation steps of the assays were performed at 37 °C and 5% CO_2_. First, cells were plated in 6-well plates and cultured overnight to obtain confluent monolayers. The next day, for the screening of the compounds’ antiviral activity, viral infection was carried out. For that, cell monolayers at 80–90% confluence were washed with phosphate-buffered saline (PBS) and infected with a multiplicity of infection (MOI) of 0.01 PFU/mL for 1 h in medium without FBS. Afterwards, the viral inoculum was removed and replaced by medium with 2% FBS containing the appropriate concentration of the compounds and controls. Cells were further incubated for 48 h. Then, aliquots of the cell supernatant were collected for viral titration/quantification. RDV was used as a positive control, while treatment with DMSO 0.1% was used as a vehicle control because it was the solvent for L87/PPS2, PZPs, and TZPs.

### 2.5. Viral Titration by Plaque Assay

Vero CCL-81 cells were plated in a 6-well plate the day before the assay. The cell monolayer was infected with the supernatants of the previous assay, each one added in 6 serial 10-fold dilutions (from 1:10 to 1:1,000,000). After allowing the virus to adsorb to the cells for 1 h, semi-solid agarose 0.5% (Invitrogen, Thermo Fisher Scientific, Waltham, MA, USA), an overlay medium, was added to immobilize the virus particles and inhibit their spread. The infected cells were then incubated at 37 °C and 5% CO_2_ for 48 h to allow plaque formation, where each plaque represents a viral infection unit. Following incubation, plaques were visualized by staining the cells with neutral red dye using a white light transilluminator (Avantor, Radnor, PA, USA). Plaques were counted, and viral titers were calculated based on dilution factors. For PZP25, a concentration–response curve was generated, and the concentration required to inhibit viral replication by 50% (IC50) was determined through non-linear regression using GraphPad Prism version 8.0.1.

### 2.6. Quantification of Viral RNA by qRT-PCR

We also evaluated SARS-CoV-2 replication in the candidate compound-treated versus non-treated cultures by measuring the number of viral RNA copies in the supernatants. For this purpose, we used the real-time reverse transcription polymerase chain reaction method (real-time qRT-PCR). This protocol employs TaqMan primers and probes specific to the gene encoding the envelope (E) protein [22]. As a quantification standard, we used a synthetic RNA molecule comprising the reference sequence of the N target, with a known number of copies (10^7^ copies/mL by IDT technologies). A concentration curve was prepared by serial dilution of the positive control from 10^6^ to 10^1^ copies/mL. Viral RNA was extracted from 140 μL of cell-free culture supernatants using a QIAamp Viral RNA mini kit (Qiagen, Hilden, Germany), according to the manufacturer’s instructions. Reverse transcription and gene amplification were performed in one-step reactions with a qRT-PCR kit developed by the Biomanguinhos Institute (Fiocruz, Rio de Janeiro, Brazil) in an ABI 7500 thermocycler (Applied Biosystems, Waltham, MA, USA).

### 2.7. Evaluation of Cellular Cytotoxicity by LDH Measurement

For evaluation of LDH levels in treated cells, Vero E6 cells were seeded at 10^4^ cells/well in 96-well plates and incubated overnight at 37 °C and 5% CO_2_. The next day, compounds were added in triplicate at respective concentrations, and cells remained incubated for 48 h. Appropriate controls as per assay instructions were added, including a maximum LDH activity control, positive and negative LDH controls, blank (0.1% DMSO), and drug controls RDV and NMV. After that, cell supernatants were used to measure LDH released with a commercial CyQUANT™ LDH Cytotoxicity Assay kit (Thermofisher). After adding specific assay solutions and controls, the microtiter plate was incubated for 30 min at room temperature, protected from light. LDH activity was measured by absorbance (OD) at 490/680 nm in a Spectramax M5 series device (Molecular Devices, San Jose, CA, USA). Cytotoxicity was calculated according to the manufacturer’s guidelines.

### 2.8. Determination of Pre- and Post-Infection Antiviral Effect

Vero E6 cells were seeded one day before the experiment. Treatment was performed in the following conditions. (i) Pre-treatment virus: PZP25 was added to virus suspensions and co-incubated for 1 h at 4 °C, 20 °C, or 37 °C, and then suspensions containing the virus and PZP25 were added to cell cultures for 1 h at 37 °C and then washed out, and cells were incubated for 48 h at 37 °C in medium without DMSO. (ii) Pre-treatment cells: PZP25 was added to the cell cultures for 1 h at 37 °C and then washed out; the virus was added for 1 h (without PZP25) at 37 °C and then washed out, and cells were incubated for 48 h at 37 °C in medium without DMSO. (iii) During treatment: PZP25 and the virus were added concomitantly to the cell cultures for 1 h at 37 °C and then washed out, and cells were incubated for 48 h at 37 °C in medium without DMSO. (iv) Post-treatment: the virus was added to cell cultures for 1 h at 37 °C and then washed out; PZP25 (in DMSO, final concentration of 0.1%) was added, and cells were incubated for 48 h at 37 °C. (v) Controls: the virus was added to the cell cultures for 1 h at 37 °C and then washed out, and either medium or medium containing DMSO 0.1% was added, and cells were incubated for 48 h at 37 °C. PZP25 was used at a concentration of 100 µM. For wash outs, following the 1 h viral adsorption at 37 °C, the viral inoculum was cautiously aspirated from the cell monolayer to remove unbound virus particles, and then the cells were carefully washed two to three times with 2 mL of pre-warmed PBS per well, ensuring not to displace the cells. Then, the wash solution was slowly poured into the edge of the well, the plate was gently agitated for 5–10 s, and the solution was completely removed each time. These washing steps eliminate extracellular virus while preserving cell integrity, followed by the addition of maintenance medium (DMEM supplemented with 2% FBS, with or without DMSO 0.1%) for 48 h. All procedures related to cell infection and the final steps of viral supernatant collection, RNA quantification, and statistical analysis were conducted as previously described.

### 2.9. Statistical Analysis

Statistical analyses were conducted using GraphPad Prism version 8.0.1 (GraphPad Software Inc., San Diego, CA, USA), employing one-way ANOVA with Dunnett’s or Sidak’s multiple comparisons tests for the concentration response assays of compounds for treatment experiments. Non-linear regression was employed to determine PZP25 IC50. The experimental results were considered significant when * *p* < 0.05, ** *p* < 0.01, *** *p* < 0.001, and **** *p* < 0.0001.

## 3. Results

### 3.1. PZP and TZP Derivatives Show In Vitro Antiviral Activity Against SARS-CoV-2

A total of twenty compounds—including the pyrazolopyridine–sulfonamide compound PPS2 [15] (also referred to as compound L87 in [14]), five PZPs (Table 1), and fourteen TZPs [20] (Table 2)—were initially screened in vitro for antiviral activity against the SARS-CoV-2 Wuhan strain. Screening was performed at a fixed concentration of 100 µM using a plaque reduction assay as the readout. Control wells containing only vehicle (DMSO 0.1%) served as the baseline, representing 100% virus growth for normalization purposes. Careful visual inspection was performed for assessment of aggregation and solubility of the compounds, and complete dissolution was apparent, with no visible precipitates or aggregates.

**Table 1:** List of pyrazolopyrimidines and pyrazolopyridine–sulfonamide (PPS2) with their chemical structure, molecular mass, and anti-SARS-CoV-2 in vitro activity (at 100 μM).

Pyrazolopyrimidines * and PPS2 **			
Chemical Structure	Code	Molecular Mass (g/mol)	SARS-CoV-2 Inhibition at 100 μM ^#^
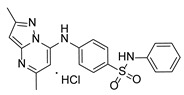	PZP25	429	100%
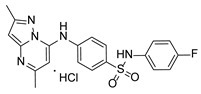	PZP27	447	100%
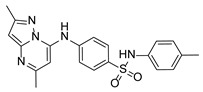	PZP28	407	100%
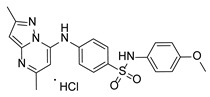	PZP29	459	100%
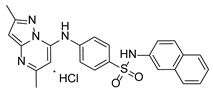	PZP31	479	No inhibition
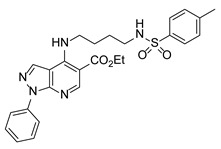	PPS2	507	45%

**Figure 1:**
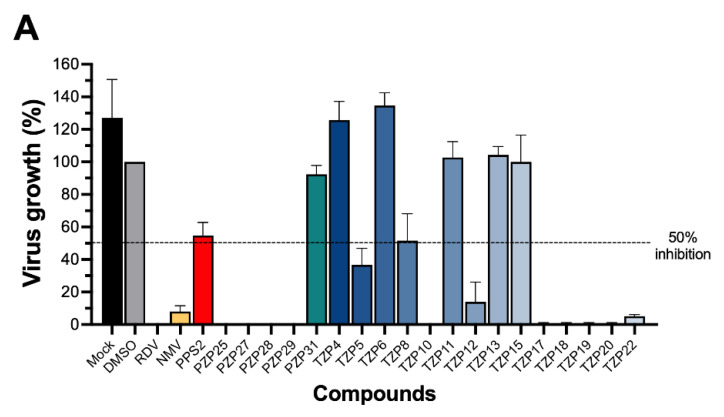

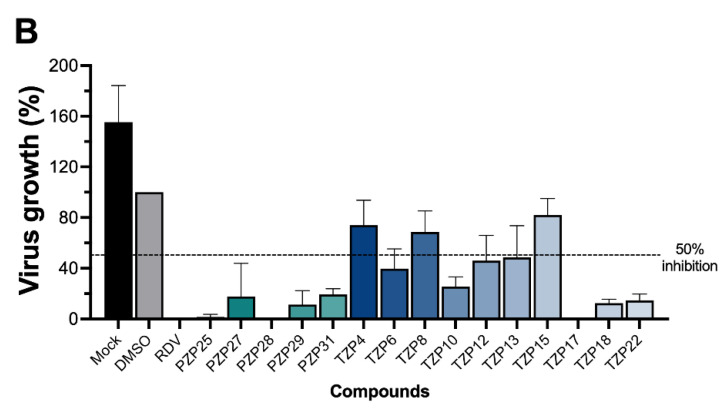
Screening of anti-SARS-CoV-2 activity for PPS2, pyrazolopyrimidines (PZPs), and triazolopyrimidines (TZPs) using an in vitro viral inhibition assay. Vero E6 cells were infected with SARS-CoV-2 at a multiplicity of infection (MOI) of 0.01. Subsequently, cells were exposed to 100 µM of compounds PPS2, five PZPs, and fourteen TZPs in a saline vehicle containing either 1% (*v*/*v*) dimethyl sulfoxide (DMSO) for panel (**A**) or 0.1% (*v*/*v*) DMSO for panel (**B**). (**A**) Anti-SARS-CoV-2 activity with 1% (*v*/*v*) DMSO and viral quantification by PFU/mL. Vehicle-only wells (1% DMSO) served as the 100% viral growth control. ‘Mock’ wells (medium only, no vehicle) were included. Antiviral drugs remdesivir (RDV) and nirmatrelvir (NMV) at 5 µM were used as positive controls. No infection (NI) wells (no virus added) served as controls. The virus was in contact with cells for one hour and was then washed out. Compounds remained in wells for 48 h. Data are shown as percent inhibition of virus growth relative to vehicle-only wells, representing the mean ± standard deviation (SD) from three independent experiments. (**B**) Anti-SARS-CoV-2 activity with 0.1% (*v*/*v*) DMSO and viral quantification by RT-PCR. Vero E6 cells were infected with SARS-CoV-2 at an MOI of 0.01 and exposed to 100 µM of compounds in saline vehicle containing 0.1% (*v*/*v*) DMSO. Due to limited amounts, five compounds (PPS2, TZP5, TZP11, TZP19, and TZP20) were not assayed in this run. Controls (vehicle, mock, RDV) and experimental timelines were as described for panel (**A**), with 0.1% DMSO in the vehicle. Viral replication was measured by quantitative reverse transcription polymerase chain reaction (RT-PCR). Data are shown as percent inhibition of virus growth relative to vehicle-only wells, representing the mean ± SD from three independent experiments. Different column colors were used to identify the different classes of molecules (black and grey for medium-only or DMSO controls, yellow for positive controls RDV and NMV, red for PSS2, green for PZPs and blue for TZPs; different green and blue shades were used for individual PZP and TZP molecules).

*: These compounds have been originally described by Silveira and coworkers [20]. **: This compound was originally described by Silva and coworkers [15]. ^#^: SARS-CoV-2 growth inhibition in plaque assays (refer to Figure 1A).

**Table 2:** List of triazolopyrimidines with their chemical structure, molecular mass, and anti-SARS-CoV-2 in vitro activity (at 100 μM).

Triazolopyrimidines *			
Chemical Structure	Code	Molecular Mass (g/mol)	SARS-CoV-2 Inhibition at 100 μM ^#^
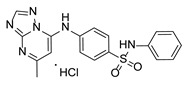	TZP4	416	No inhibition
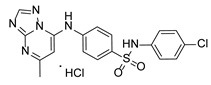	TZP5	450	63%
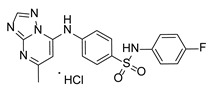	TZP6	434	No inhibition
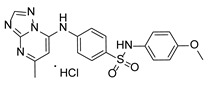	TZP8	446	50%
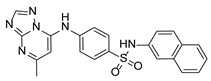	TZP10	430	100%
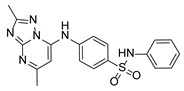	TZP11	394	No inhibition
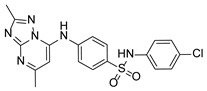	TZP12	428	86%
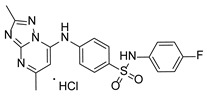	TZP13	448	No inhibition
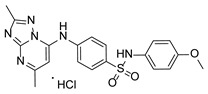	TZP15	460	No inhibition
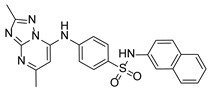	TZP17	444	>99%
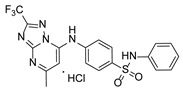	TZP18	484	>99%
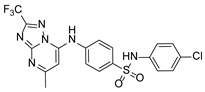	TZP19	482	>99%
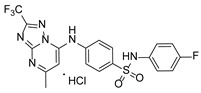	TZP20	502	>99%
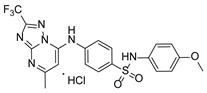	TZP22	514	95%

*: These compounds were originally described by Silveira et al. [20]. ^#^: SARS-CoV-2 growth inhibition in plaque assays (refer to Figure 1A).

Twelve out of the twenty tested compounds (PZPs 25, 27, 28, and 29; TZPs 5, 10, 12, 17, 18, 19, 20, and 22) demonstrated moderate to strong antiviral activity, reducing viral replication by a range of 65% to 100% relative to the vehicle control (Figure 1A). Conversely, one PZP (PZP31), six TZPs (TZPs 4, 6, 8, 11, 13, and 15), and the PPS2 compound exhibited limited or no antiviral effect (<50% inhibition). The positive controls, RDV and NMV, resulted in near-complete suppression of viral replication, validating assay performance.

A subsequent screening round was performed to confirm and quantify viral inhibition. For this, newly synthesized lots of each compound were prepared and purified (>95% purity), and a different virus growth detection method (quantitative RT-PCR) was used. Quality amounts of five compounds (PPS2, TZP5, TZP11, TZP19, and TZP20) were not obtained in this second round, and, therefore, confirmatory screening was only performed with the other 15 compounds. Overall, the results corroborated the findings from the plaque assay, with comparable inhibitory profiles across most compounds (Figure 1B). The only notable discrepancy was observed for compound PZP31, which displayed minimal antiviral activity in the plaque assay but demonstrated measurable inhibition in the RT-qPCR-based assay, suggesting a possible difference in detection sensitivity between methodologies or compound kinetics.

**Figure 2:**
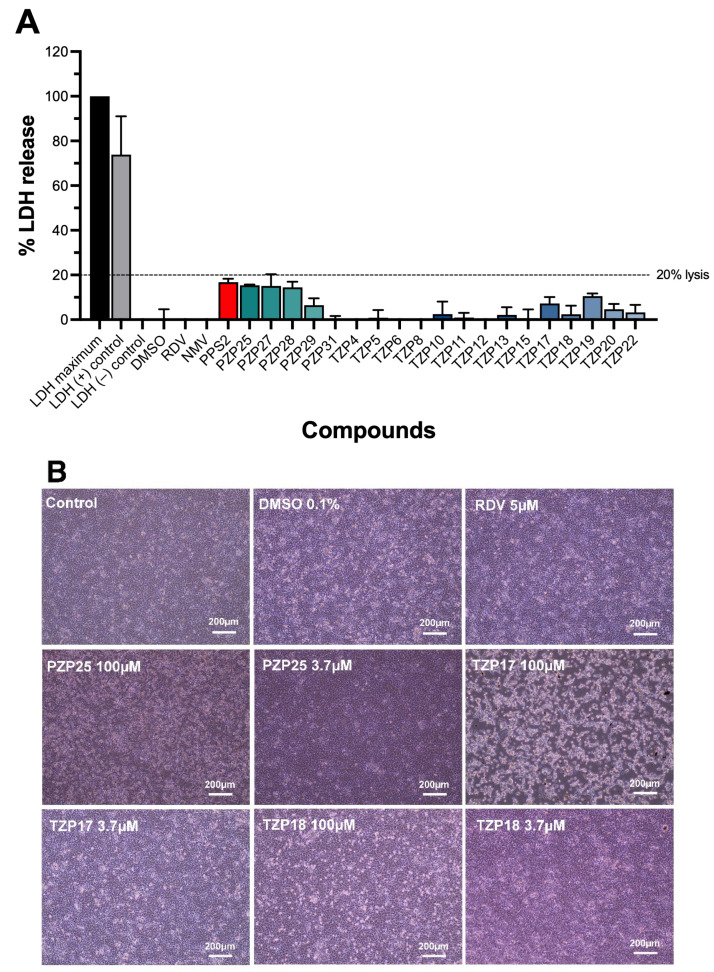
Cytotoxicity and morphological evaluation of compounds PPS2, pyrazolopyrimidines (PZPs), and triazolopyrimidines (TZPs) in Vero E6 cells. (**A**) Cytotoxicity was assessed via lactate dehydrogenase (LDH) release assay. Vero E6 cells were exposed to 100 µM of PPS2, five PZPs, and fourteen TZPs. Controls included saline vehicle with 1% (*v*/*v*) dimethyl sulfoxide (DMSO), remdesivir (RDV), nirmatrelvir (NMV) at 5 µM, and appropriate maximum LDH release controls. LDH release in cell supernatants was measured using a commercial CyQUANT™ LDH Cytotoxicity Assay kit (Thermofisher). Data are expressed as the percentage of LDH released, normalized to the maximum LDH release control. Results represent the mean ± standard deviation (SD) from three independent experiments. Different column colors were used to identify the different classes of molecules (black and grey for medium-only or DMSO controls, yellow for positive controls RDV and NMV, red for PSS2, green for PZPs and blue for TZPs; different green and blue shades were used for individual PZP and TZP molecules). (**B**) Microscopic examination of Vero E6 cells exposed for 48 h to medium without DMSO or any compounds, medium containing DMSO 0.1%, remdesivir (RDV) 5 µM, and compounds PZP25, TZP17, and TZP18 at 3.7 and 100 µM. All exposed cells showed little or no morphological alterations, except for cells exposed to TZP17 100 µM, which induced evident loss of confluence of the monolayer and caused detachment with floating cells (the latter not shown in the picture but visualized by focal distance adjustments). Magnification: 100×.

No measurable cytotoxicity was detected in Vero E6 cells treated with any of the tested compounds at 100 µM, as indicated by the absence of significant LDH release (Figure 2A). LDH levels in treated cells remained comparable to untreated controls (*p* > 0.05 for all compounds vs. control). Active compounds PZP25, TZP17, and TZP18 were selected for a closer microscopical examination. Vero E6 cells incubated with compounds PZP25 and TZP18 showed no evident morphological changes at concentrations up to 100 µM. On the other hand, TZP17 showed evident cell detachment and loss of confluence at 100 µM (Figure 2B).

### 3.2. Preliminary Structure–Activity Relationship

The chemical structures of the PZP and the TZP compounds used in this study are described in Table 1 and Table 2. The 14 TZPs belong to either of three subclasses based on the substitute added in position 2 of the [1,2,4]triazolo[1,5-a]pyrimidine nucleus: (1) [1,2,4]triazolo[1,5-a]pyrimidine nucleus with a hydrogen as substitute (compounds **4**–**6**, **8**, and **10**); (2) [1,2,4]triazolo[1,5-a]pyrimidine nucleus with a methyl group (compounds **11**–**13**, **15**, and **17**); and (3) [1,2,4]triazolo[1,5-a]pyrimidine nucleus with a trifluoromethyl group (compounds **18**–**20** and **22**). The PZPs (compounds **25**, **27**–**29**, and **31**) are similar to the subclass 2 (methyl group) of TZPs. Each compound in each subclass varies according to a substitute group in the benzenesulfonamide moiety, which can be either hydrogen (-H), methyl (-CH3), chlorine (-Cl), fluorine (-F), methoxy (-O-CH3), or a second benzene structure forming a naftalene. Among the TZPs, the ones with the best performances in inhibiting SARS-CoV-2 growth at 100 μM belonged to the subclass 3 (CF3 group linked to the [1,2,4]triazolo[1,5-a]pyrimidine nucleus). Among the TZPs of the other two subclasses (1 and 2), only the compounds with a naftalene substitution (TZPs 10 and 17) showed strong activity, but compound TZP17 also induced loss of confluence and detachment of Vero E6 cells. All five PZP compounds showed strong activity at 100 μM.

### 3.3. PZP25 Exhibits Antiviral Activity and Low Cytotoxicity Against SARS-CoV-2

**Figure 3:**
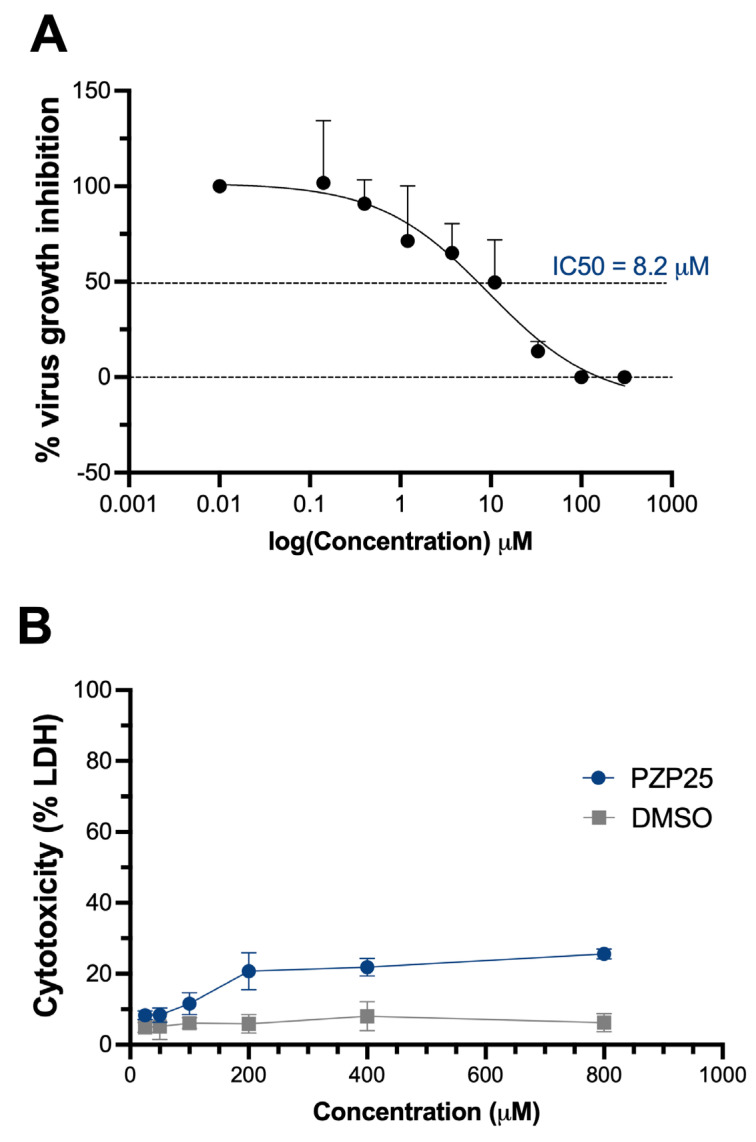
Determination of the 50% inhibitory concentration (IC50) and 50% cytotoxic concentration (CC50) for compound PZP25. (**A**) Determination of IC50 of PZP25 against SARS-CoV-2. Vero E6 cells were infected with SARS-CoV-2 at a multiplicity of infection (MOI) of 0.01. Cells were then exposed to PZP 25 at concentrations ranging from 0.14 µM to 300 µM, prepared in 3-fold serial dilutions. Saline vehicle containing 0.1% (*v*/*v*) dimethyl sulfoxide (DMSO) served as the 100% viral growth control. Following a one-hour virus adsorption period, the inoculum was removed by washing, and compounds remained in the wells for 48 h. Viral replication was measured by quantitative reverse transcription polymerase chain reaction (RT-PCR). Data are presented as the percentage inhibition of virus growth relative to vehicle-only wells, representing the mean ± standard deviation (SD) from three independent experiments. The IC50 value was calculated using non-linear regression analysis in GraphPad Prism software. (**B**) Determination of CC50 of PZP25. Vero E6 cells were exposed to PZP25 at concentrations ranging from 25 µM to 800 µM, prepared in 2-fold serial dilutions. Controls included saline vehicle containing 0.03–1.0% (*v*/*v*) DMSO, as well as appropriate maximum LDH release controls. After a 48 h incubation period, cell supernatants were collected, and lactate dehydrogenase (LDH) release was measured using a commercial CyQUANT™ LDH Cytotoxicity Assay kit (Thermofisher). Data are shown as the percentage of LDH released by cells, normalized to the maximum LDH release control, and represent the mean ± SD from three independent experiments.

Our initial high-throughput screening identified several PZP and TZP compounds that demonstrated robust antiviral activity, achieving nearly complete inhibition of SARS-CoV-2 replication (approximately 100% virus growth inhibition) at a concentration of 100 µM. To further characterize these promising candidates, one compound, PZP25, which displayed potent activity during the initial screen, was selected for detailed half-maximal inhibitory concentration (IC50) and half-maximal cytotoxic concentration (CC50) determinations. PZP25 exhibited an IC50 of 8.2 µM against SARS-CoV-2 replication (Figure 3A). Importantly, the compound demonstrated no significant cytotoxicity in Vero E6 cells up to the highest concentration tested (800 µM) (Figure 3B). For a precise determination of the selectivity index (SI), higher concentrations needed to be tested, but this data reveals an SI above 100, which indicates that the compound has a wide margin of safety.

### 3.4. Continued Exposure to PZP25 Is Required for Potent Antiviral Activity

**Figure 4:**
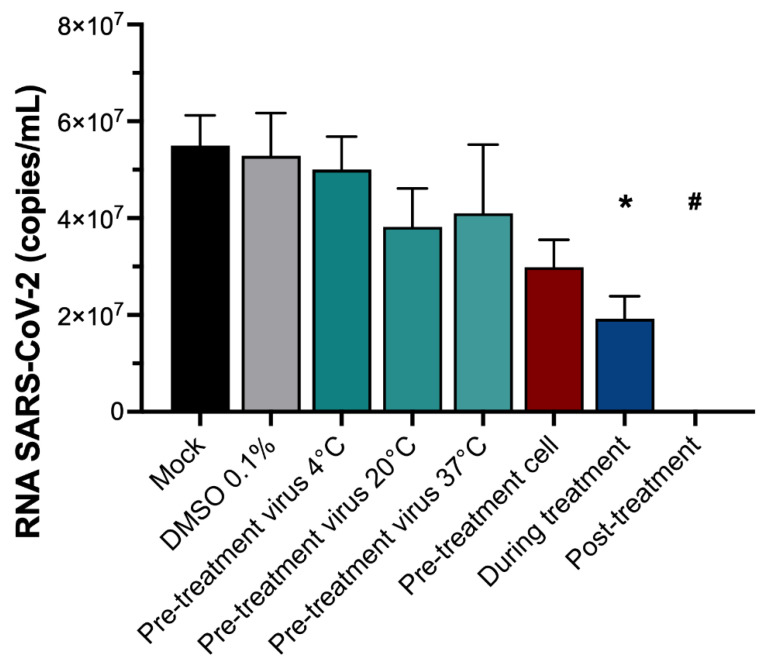
Time of addition evaluation of PZP25 against SARS-CoV-2. SARS-CoV-2-infected Vero E6 cells were treated with different treatment schemes using PZP25 at 100 µM. The assay conditions were as follows. (i) Pre-treatment virus: PZP25 was added to virus suspensions and co-incubated for 1 h at 4 °C, 20 °C, or 37 °C, and then suspensions containing the virus and PZP25 were added to cell cultures for 1 h at 37 °C and were washed out, and cells were incubated for 48 h at 37 °C in medium without DMSO. (ii) Pre-treatment cells: PZP25 was added to the cell cultures for 1 h at 37 °C and then washed out; the virus was added for 1 h (without PZP25) at 37 °C and was washed out, and cells were incubated for 48 h at 37 °C in medium without DMSO. (iii) During treatment: PZP25 and the virus were added concomitantly to the cell cultures for 1 h at 37 °C and were washed out, and cells were incubated for 48 h at 37 °C in medium without DMSO. (iv) Post-treatment: the virus was added to cell cultures for 1 h at 37 °C and was washed out; PZP25 (in DMSO, final concentration of 0.1%) was added, and cells were incubated for 48 h at 37 °C. (v) Controls: the virus was added to the cell cultures for 1 h at 37 °C and was washed out, and either medium (NT/I) or medium containing DMSO 0.1% was added, and cells were incubated for 48 h at 37 °C. Viral titers were quantified in cell supernatants collected at 48 h post-infection. Viral replication was measured by quantitative reverse transcription polymerase chain reaction (RT-PCR). Each bar represents a specific treatment condition. Virus growth under the different conditions is expressed as the number of SARS-CoV-2 RNA copies per mL. Statistical analysis was performed using one-way ANOVA with Dunnett’s multiple comparisons test (pre-treatment and during treatment conditions versus mock; * *p* = 0.0254) or a *t*-test (post-treatment condition versus DMSO 0.1%; # *p* = 0.0266). *n* = 3 (number of independent replicates for each experiment). Mock: medium only, no vehicle, non-treated infected control.

To elucidate the putative mechanism of action of PZP25 against SARS-CoV-2, we conducted a ToA assay in vitro. A concentration of 100 µM PZP25 was used, selected based on prior dose–response experiments to ensure robust antiviral effects. Our results demonstrated that continuous presence of PZP25 throughout the assay (designated ‘post-treatment’) completely inhibited SARS-CoV-2 replication, with viral RNA levels reduced to undetectable levels (*p* = 0.0266 compared to DMSO 0.1% control) (Figure 4).

Treatment applied solely during the 1 h virus–cell incubation period (‘during treatment’) significantly reduced viral replication but did not eliminate it. Viral RNA levels decreased to approximately ~65% in relation to the untreated control (*p* = 0.0254 compared to ‘mock’) (Figure 4).

On the other hand, pre-treatment conditions showed no effectiveness. Pre-incubation of the virus with PZP25 at 4 °C, 20 °C, and 37 °C prior to cell infection did not significantly alter viral RNA levels compared to the untreated control. Although not significantly decreasing virus growth, the pre-treatment conditions at 20 °C and 37 °C may have affected it (apparent lower mean growth and more variation, with wider standard deviations) (Figure 4). These data suggest that pre-incubation of the drug with the virus actually decreases the antiviral activity of PZP25 (because the ‘during treatment’ condition, which does not have a virus compound pre-incubation step, showed stronger antiviral activity). Pre-treating host cells with PZP25 before infection and subsequent washout (‘pre-treatment cell’) also did not result in a significant reduction in viral RNA levels. Therefore, the results indicate that PZP25’s inhibitory action depends on its presence at an effective concentration during the interaction of the virus with the host cell, and a complete growth inhibitory effect is achieved with continuous presence of the compound during incubation.

## 4. Discussion

The persistent global public health challenge posed by SARS-CoV-2 underscores an urgent need for novel antiviral strategies beyond existing vaccines and therapeutics [4,6]. In this study, we aimed to identify new chemical scaffolds with potent anti-SARS-CoV-2 activity by screening a series of PZP and TZP derivatives, building upon prior molecular docking and dynamics insights [15] and their established antimalarial properties [15,20]. Our findings reveal that PZPs and TZPs represent a promising class of compounds with significant inhibitory activity against SARS-CoV-2 in vitro, offering a new avenue for antiviral drug discovery.

Our initial screening demonstrated that a substantial proportion of the tested PZP and TZP compounds (12 out of 20) exhibited potent anti-SARS-CoV-2 activity, reducing viral replication by 65% to 100% at a concentration of 100 µM, without inducing cytotoxicity in Vero E6 cells. This broad activity highlights the intrinsic antiviral potential within these chemical classes. Interestingly, our previous work showed that TZPs with a CF3 moiety attached to the triazolo ring were highly effective against *Plasmodium falciparum*, a correlation we also observed in the present study for SARS-CoV-2 inhibition. Conversely, while PZPs displayed only moderate antiplasmodial activity, they emerged as strong inhibitors of SARS-CoV-2 replication. This differential activity profile suggests distinct or overlapping mechanisms of action against diverse pathogens, positioning the PZP and TZP scaffolds as versatile platforms for developing targeted or broad-spectrum agents.

One aspect of our investigation involved validating in silico predictions with in vitro experimental data. A pyrazolopyridine–sulfonamide derivative, L87/PPS2, previously shown by Daoud and coworkers [14] to possess high theoretical affinity for SARS-CoV-2 targets—even surpassing remdesivir in docking simulations—exhibited only partial inhibitory effects (approximately 50% at 100 µM) in our in vitro assays. This significant discrepancy between computational predictions and experimental outcomes underscores the critical importance of empirical validation in drug discovery. While in silico methods are invaluable for identifying promising candidates, factors such as cell permeability, metabolic stability, protein binding in complex biological environments, and the dynamic nature of drug–target interactions in vivo often influence actual biological activity, which cannot be fully captured by static docking models alone. This finding redirected our focus towards the more potent PZP and TZP derivatives for further development.

Preliminary structure–activity relationship analysis provided key insights into the molecular features driving anti-SARS-CoV-2 efficacy. For TZPs, the presence of a CF3 group at position 2 of the triazolopyrimidine nucleus was consistently associated with strong antiviral activity. Additionally, TZPs lacking the CF3 group but possessing a naphthalene moiety in the benzenesulfonamide portion also demonstrated robust inhibition. These observations suggest two independent structural determinants for activity within the TZP class. A logical next step for lead optimization would be the rational synthesis and evaluation of hybrid molecules incorporating both the CF3 and naphthalene moieties. This combinatorial approach could potentially yield compounds with enhanced potency and improved pharmacokinetic properties, representing a compelling future research direction.

Conversely, the preliminary structure–activity relationship for PZPs presented an intriguing, seemingly contradictory pattern compared to TZPs. While all five tested PZPs showed strong activity at 100 µM, the naphthalene group, which enhanced TZP activity, appeared to decrease the inhibitory effect in PZP31, making it the least active PZP compound. This divergence strongly suggests that PZPs and TZPs, despite their structural similarities, likely interact with distinct viral or host targets or bind to similar targets via different modes. Further in silico studies, such as molecular dynamics simulations and binding energy calculations, focusing on specific SARS-CoV-2 protein targets (e.g., 3CLpro, RdRp, helicase, or even host factors), would be instrumental in deciphering these differential binding preferences. Comprehensive elucidation of these structure–activity relationships is crucial for the rational design of highly optimized antiviral agents within these scaffolds.

Among the most active compounds, PZP25 was selected for in-depth characterization due to its potent activity during the initial screen. PZP25 demonstrated an IC50 of 8.2 µM against SARS-CoV-2 replication, coupled with a high CC50 of >800 µM. Although an IC50 in the low micromolar range is a common starting point in early drug discovery, the remarkable selectivity indicates a favorable therapeutic window and low general cellular toxicity, which is a critical attribute for any promising drug candidate. Future experiments will prioritize determining the IC50 and CC50 values for all other highly active PZP and TZP compounds to identify the lead candidates with the most optimal balance of potency and safety for subsequent preclinical development. The high CC50 values observed for PZP25 indicate low overt cytotoxicity under the assay conditions, suggesting a favorable preliminary safety profile. While this does not exclude the possibility of off-target effects, the lack of detectable cytotoxicity at concentrations well above the antiviral IC50 argues against major nonspecific cellular toxicity.

To elucidate the mechanism of action of PZP25, time of addition (ToA) experiments were performed. The protocols of pre-treatment either of the virus (at different temperatures) or of the cells with the compound failed to result in significant inhibitions of virus growth, indicating that the compound does not exert irreversible virucidal activity or permanently inactivate viral particles prior to cell contact. Interestingly, a partial (~60%) yet significant inhibition was observed during the 1 h virus–cell incubation period (‘during treatment’). Because the ‘during treatment’ condition does not have a virus/compound pre-incubation step, these data suggest that pre-incubation of the drug with the virus actually decreases the antiviral activity of PZP25. The mechanism behind this effect is unknown and deserves further investigation. Complete inhibition of virus growth was observed in the post-treatment condition, when PZP25 was continuously present. Therefore, the action of the compound takes place during the interaction of the virus with the host cells. Because substantial, although partial, inhibition occurred in the 1 h ‘during treatment’ condition, it indicates that it affects the first cycle of viral replication, but continued exposure to the compound is necessary to inhibit secondary replication cycles of viruses that escaped the first cycle inhibition. In any case, the assay does not allow for determining specific inhibitory mechanisms, such as blockade of virus entry into the cell or post-entry inhibitory mechanisms. In silico studies showed that the target of L87/PPS2 was the main protease of SARS-CoV-2, 3CLpro [14], an enzyme that mediates the proteolytic processing of two large virus replicase polyproteins, generating 16 structural proteins [23]. The target of PZP25 is yet unknown, but given its structural similarities (pyrazolopyrimidine–sulfonamide) with PPS2 (a pyrazolopyridine–sulfonamide), its target may as well be 3CLpro, and it may act at post-entry stages of the viral life cycle. Following this initial and promising screening, studies leading to the determination of the most potent PZP and/or TZP compound against SARS-CoV-2, followed by mechanistic experiments, such as intracellular viral RNA kinetics over time and biochemical inhibition assays, for instance, with 3CLpro, will be needed to determine the mechanism of action. Of note, the necessity for sustained compound contact, as highlighted by the ‘post-treatment’ efficacy, has critical implications for further drug design, emphasizing the need for favorable pharmacokinetic profiles to maintain therapeutic concentrations in vivo.

None of the tested compounds at 100 μM showed substantial cytotoxicity activity measured by LDH release, and three of them (PZP25, TZP17, and TZP18) were subjected to closer microscopical examination. Contrary to PZP25 and TZP18, the compound TZP17 induced evident loss of confluence, even in virus-negative cell monolayers at 100 μM, but LDH release was still kept well below 20%. This uncoupling of morphological changes from overt cell death suggests a specific interaction with host cell physiology or host–pathogen interactions that do not immediately lead to cell lysis. In the case of infected cell cultures, the subtle alterations could reflect interference with viral budding, cell-to-cell viral spread, cytoskeletal dynamics, or cell adhesion molecules, which are often manipulated by viruses. Further detailed investigation into these subtle cellular effects, utilizing techniques such as proteomics and transcriptomics, could uncover novel host targets or pathways modulated by these compounds, providing additional avenues for therapeutic intervention.

Although the antiviral activities of PZPs and TZPs shown are promising, this study has a number of limitations. As discussed above, the mechanisms of viral growth inhibition by these compounds need to be determined, as well as their activities in variants of SARS-CoV-2 and in other types of host cells. Vero E6 cells were selected for this initial study because they are highly permissive to SARS-CoV-2 infection, widely used for antiviral screening, and allow robust and reproducible measurements of viral replication. However, there are limitations, including the lack of a fully competent interferon response and differences in cellular pathways compared to human airway epithelial cells. Therefore, validation in human respiratory cell models, such as Calu-3 or primary human airway epithelial cultures, will be an important next step to better assess translational relevance and host-dependent antiviral effects.

## 5. Conclusions

In conclusion, this study successfully identified novel PZP and TZP derivatives with potent anti-SARS-CoV-2 activity in vitro and favorable selectivity profiles. The characterization of PZP25 revealed a promising lead compound with low micromolar potency and high selectivity, primarily acting at post-entry stages of the viral life cycle. The preliminary structure–activity relationship findings offer a foundation for rational drug design and optimization within these chemical scaffolds. Future research should focus on precisely elucidating the molecular targets of these compounds, optimizing their pharmacokinetic properties, evaluating their efficacy against SARS-CoV-2 variants of concern, evaluating combination with current anti-SARS-CoV-2 drugs, and, ultimately, assessing their in vivo antiviral activity and safety. The identification of these novel chemical scaffolds represents a significant step towards developing effective new antiviral agents to combat current and future coronavirus threats.

## Data Availability

The data that support the findings of this study are available from the corresponding author upon reasonable request. Some data may not be made available because of privacy or ethical restrictions.

## Abbreviations

The following abbreviations are used in this manuscript:

SARS-CoV-2	Severe acute respiratory syndrome coronavirus 2
COVID-19	Coronavirus disease 2019
PPS2	Pyrazolopyridine–sulfonamide compound 2
PZP	Pyrazolopyrimidine
TZP	Triazolopyrimidine
IC50	Inhibitory concentration 50%
CC50	Cytotoxic concentration 50%
ToA	Time of addition assay
FDA	Food and Drug Administration
RNA	Ribonucleic acid
DMSO	Dimethyl sulfoxide
RDV	Remdesivir
NMV	Nirmatrelvir
FBS	Fetal bovine serum
PCR	Polymerase chain reaction
BSL3	Biosafety level 3
WHO	World Health Organization
PBS	Phosphate-buffered saline
MOI	Multiplicity of infection
PFUs	Plaque-forming units
LDH	Lactate dehydrogenase
h	Hour
